# Supplementary material for: Comparison of sagittal plane gait characteristics between the overground and treadmill approach for gait analysis in typically developing children
Source: PeerJ. 2022 Jul 22;10:e13752. doi: 10.7717/peerj.13752 (PMC9310770; doi:10.7717/peerj.13752)
Supplement: Data S1 [file peerj-10-13752-s001.docx]

|  | | **all** | | **3-6yrs** | **7-8yrs** | **9-10yrs** | **11-12yrs** | **>13yrs** |
| --- | --- | --- | --- | --- | --- | --- | --- | --- |
| **n** | 57 | | 12 | | 10 | 16 | 11 | 8 |
| **Age (yrs)** | 9.28  (2.88 ; 15.68) | | 4.83  (2.49 ; 7.17) | | 7.40  (6.39 ; 8.41) | 9.56  (8.56 ; 10.57) | 11.6  (10.65 ; 12.63) | 14.50  (11.19 ; 17.81) |
| **Weight (kg)** | 35.29  (4.52 ; 66.06) | | 19.88  (12.73 ; 27.02) | | 25.56  (19.14 ; 31.98) | 33.65  (22.46 ; 44.83) | 46.26  (19.02 ; 73.51) | 58.76  (29.43 ; 88.09) |
| **Height (cm)** | 1.40  (0.99 ; 1.81) | | 1.12  (0.86 ; 1.39) | | 1.28  (1.20 ; 1.36) | 1.43  (1.30 ; 1.55) | 1.57  (1.32 ; 1.81) | 1.68  (1.50 ; 1.86) |
| **leg length (cm)** | 0.73  (0.48 ; 0.97) | | 0.56  (0.39 ; 0.72) | | 0.67  (0.59 ; 0.76) | 0.75  (0.66 ; 0.83) | 0.83  (0.70 ; 0.95) | 0.88  (0.81 ; 0.95) |
