# Supplementary material for: Comparison of sagittal plane gait characteristics between the overground and treadmill approach for gait analysis in typically developing children
Source: PeerJ. 2022 Jul 22;10:e13752. doi: 10.7717/peerj.13752 (PMC9310770; doi:10.7717/peerj.13752)
Supplement: Supplemental Information 12 [file peerj-10-13752-s012.docx]

**Statistical analysis**

1. **Test normal distribution of data with Kolmogorov-Smirnov test**
   1. **Spatiotemporal parameters**

_V = Overground approach

_C = Treadmill approach

| **Tests of Normality** | | | | | | |
| --- | --- | --- | --- | --- | --- | --- |
|  | Kolmogorov-Smirnov^a^ | | | Shapiro-Wilk | | |
|  | Statistic | df | Sig. | Statistic | df | Sig. |
| Speed_V | ,091 | 57 | ,200^*^ | ,958 | 57 | ,046 |
| Speed_C | ,096 | 57 | ,200^*^ | ,946 | 57 | ,013 |
| Stridetime_V | ,077 | 57 | ,200^*^ | ,982 | 57 | ,551 |
| Stridetime_C | ,051 | 57 | ,200^*^ | ,989 | 57 | ,889 |
| Stance_V | ,076 | 57 | ,200^*^ | ,929 | 57 | ,003 |
| Stance_C | ,070 | 57 | ,200^*^ | ,987 | 57 | ,809 |
| Swing_V | ,098 | 57 | ,200^*^ | ,907 | 57 | ,000 |
| SwingR_C | ,099 | 57 | ,200^*^ | ,985 | 57 | ,701 |
| Stepwidth_V_cm | ,164 | 57 | ,001 | ,864 | 57 | ,000 |
| Stepwidth_C_cm | ,149 | 57 | ,003 | ,963 | 57 | ,082 |
| Steplength_Vcm | ,089 | 57 | ,200^*^ | ,962 | 57 | ,068 |
| Steplength_Ccm | ,073 | 57 | ,200^*^ | ,981 | 57 | ,486 |
| *. This is a lower bound of the true significance. | | | | | | |
| a. Lilliefors Significance Correction | | | | | | |

- Based on Sig. Of the Kolmogorov-Smirnov test, we assume that this data is normal distributed, except step width.
  1. **Peaks in sagittal joint angles**

| **Tests of Normality** | | | | | | |
| --- | --- | --- | --- | --- | --- | --- |
|  | Kolmogorov-Smirnov^a^ | | | Shapiro-Wilk | | |
|  | Statistic | df | Sig. | Statistic | df | Sig. |
| VavgPelvTilt | ,107 | 57 | ,159 | ,977 | 57 | ,347 |
| CavgPelvTilt | ,114 | 57 | ,060 | ,976 | 57 | ,324 |
| CPeakHipExt | ,078 | 57 | ,200^*^ | ,984 | 57 | ,633 |
| CPeakHipFLexTS | ,071 | 57 | ,200^*^ | ,965 | 57 | ,094 |
| CStancePhaseKneeFlexion | ,086 | 57 | ,200^*^ | ,981 | 57 | ,514 |
| CkneeExtension_MS | ,060 | 57 | ,200^*^ | ,989 | 57 | ,892 |
| CMaxKneeFlex_Swing | ,062 | 57 | ,200^*^ | ,987 | 57 | ,806 |
| CPeakDorsalFlex | ,097 | 57 | ,200^*^ | ,983 | 57 | ,592 |
| CPeakPlantarFlex | ,088 | 57 | ,200^*^ | ,989 | 57 | ,881 |
| VPeakHipExt | ,084 | 57 | ,200^*^ | ,953 | 57 | ,027 |
| VPeakHipFLexTS | ,110 | 57 | ,081 | ,953 | 57 | ,026 |
| VStancePhasekneeflexion | ,089 | 57 | ,200^*^ | ,957 | 57 | ,043 |
| VkneeExtension_MS | ,111 | 57 | ,077 | ,950 | 57 | ,019 |
| VMaxKneeFlexionSwing | ,113 | 57 | ,066 | ,955 | 57 | ,035 |
| VPeakDorsalFlex | ,061 | 57 | ,200^*^ | ,961 | 57 | ,066 |
| VPeakPlantarFlex | ,069 | 57 | ,200^*^ | ,989 | 57 | ,875 |
| *. This is a lower bound of the true significance. | | | | | | |
| a. Lilliefors Significance Correction | | | | | | |

- 1. **Peaks in GRFs**

| **Tests of Normality** | | | | | | |
| --- | --- | --- | --- | --- | --- | --- |
|  | Kolmogorov-Smirnov^a^ | | | Shapiro-Wilk | | |
|  | Statistic | df | Sig. | Statistic | df | Sig. |
| ViconMaxPost | ,114 | 56 | ,068 | ,947 | 56 | ,015 |
| ViconMaxAnt | ,091 | 56 | ,200^*^ | ,964 | 56 | ,094 |
| ViconPeak1 | ,106 | 56 | ,181 | ,941 | 56 | ,008 |
| Viconthough | ,096 | 56 | ,200^*^ | ,953 | 56 | ,028 |
| ViconPeak2 | ,087 | 56 | ,200^*^ | ,985 | 56 | ,691 |
| MaxPostCaren | ,098 | 56 | ,200^*^ | ,942 | 56 | ,009 |
| maxAntCaren | ,060 | 56 | ,200^*^ | ,990 | 56 | ,910 |
| Peak1Caren | ,124 | 56 | ,033 | ,906 | 56 | ,000 |
| thoughCaren | ,071 | 56 | ,200^*^ | ,976 | 56 | ,324 |
| Peak2Caren | ,097 | 56 | ,200^*^ | ,976 | 56 | ,339 |
| *. This is a lower bound of the true significance. | | | | | | |
| a. Lilliefors Significance Correction | | | | | | |

- 1. **Peaks in sagittal joint moments**

| **Tests of Normality^a^** | | | | | | |
| --- | --- | --- | --- | --- | --- | --- |
|  | Kolmogorov-Smirnov^a^ | | | Shapiro-Wilk | | |
|  | Statistic | df | Sig. | Statistic | df | Sig. |
| PeakHipExtM1 | ,062 | 51 | ,200^*^ | ,988 | 51 | ,897 |
| PeakHipFlexM | ,129 | 51 | ,033 | ,964 | 51 | ,125 |
| PeakAnklePFMoment | ,103 | 51 | ,200^*^ | ,925 | 51 | ,003 |
| PeakHipExtM1Caren | ,059 | 51 | ,200^*^ | ,990 | 51 | ,948 |
| PeakHipFlexMCAREN | ,092 | 51 | ,200^*^ | ,978 | 51 | ,474 |
| PeakHipExtMCaren | ,100 | 51 | ,200^*^ | ,972 | 51 | ,269 |
| PeakAnklePFMCaren | ,071 | 51 | ,200^*^ | ,988 | 51 | ,866 |
| PeakKneeExtMoment | ,147 | 51 | ,008 | ,763 | 51 | ,000 |
| PeakKneeExtCAREN | ,107 | 51 | ,200^*^ | ,971 | 51 | ,234 |
| *. This is a lower bound of the true significance. | | | | | | |
| a. Lilliefors Significance Correction | | | | | | |
|  | | | | | | |

1. **Paired sample t-test (normal distributed data) or Wilcoxon Signed Rank test (not normal distributed data) to test differences in spatiotemporal and peak parameters between the two approaches**
   1. **Spatiotemporal parameters**

| **Paired Samples Statistics** | | | | | | | | | | | |
| --- | --- | --- | --- | --- | --- | --- | --- | --- | --- | --- | --- |
|  | | | | Mean | | N | | Std. Deviation | | Std. Error Mean | |
| Pair 1 | | Speed_V | | 1,29232 | | 57 | | ,132923 | | ,017606 | |
|  |  | Speed_C | | 1,26877 | | 57 | | ,134125 | | ,017765 | |
| Pair 2 | | Stridetime_V | | ,93368 | | 57 | | ,099807 | | ,013220 | |
|  |  | Stridetime_C | | ,91491 | | 57 | | ,098762 | | ,013081 | |
| Pair 3 | | Stance_V | | ,58077 | | 57 | | ,081800 | | ,010835 | |
|  |  | Stance_C | | ,52930 | | 57 | | ,067582 | | ,008951 | |
| Pair 4 | | Swing_V | | ,35291 | | 57 | | ,043297 | | ,005735 | |
|  |  | Swing_C | | ,38526 | | 57 | | ,034388 | | ,004555 | |
| Pair 5 | | Steplength_V | | ,60368 | | 57 | | ,080506 | | ,010663 | |
|  |  | Steplength_C | | ,58158 | | 57 | | ,082587 | | ,010939 | |
| Pair 6 | StepwidthR_V_cm | | 11,4453 | | 57 | | 3,20112 | | ,42400 | |  |
|  | StepwidthR_C_cm | | 14,7368 | | 57 | | 4,17700 | | ,55326 | |  |

| **Paired Samples Correlations** | | | | |
| --- | --- | --- | --- | --- |
|  | | N | Correlation | Sig. |
| Pair 1 | Speed_V & Speed_C | 57 | ,930 | ,000 |
| Pair 2 | Stridetime_V & Stridetime_C | 57 | ,938 | ,000 |
| Pair 3 | Stance_V & Stance_C | 57 | ,833 | ,000 |
| Pair 4 | Swing_V & SwingR_C | 57 | ,529 | ,000 |
| Pair 5 | Steplength_V & Steplength_C | 57 | ,941 | ,000 |

| **Paired Samples Test** | | | | | | | | | |
| --- | --- | --- | --- | --- | --- | --- | --- | --- | --- |
|  | | Paired Differences | | | | | t | df | Sig. (2-tailed) |
|  |  | Mean | Std. Deviation | Std. Error Mean | 95% Confidence Interval of the Difference | |  |  |  |
|  |  |  |  |  | Lower | Upper |  |  |  |
| Pair 1 | Speed_V - Speed_C | ,023544 | ,049914 | ,006611 | ,010300 | ,036788 | 3,561 | 56 | ,001 |
| Pair 2 | Stridetime_V - StridetimeR_C | ,018767 | ,035001 | ,004636 | ,009480 | ,028054 | 4,048 | 56 | ,000 |
| Pair 3 | Stance_V - StanceR_C | ,051467 | ,045281 | ,005998 | ,039453 | ,063482 | 8,581 | 56 | ,000 |
| Pair 4 | Swing_V - Swing_C | -,032349 | ,038513 | ,005101 | -,042568 | -,022130 | -6,342 | 56 | ,000 |
| Pair 5 | Steplength_V - Steplength_C | ,022098 | ,028140 | ,003727 | ,014632 | ,029565 | 5,929 | 56 | ,000 |


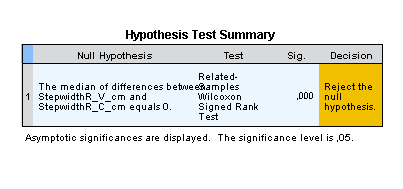


- 1. **Peaks in sagittal joint angles**

| **Paired Samples Statistics** | | | | | |
| --- | --- | --- | --- | --- | --- |
|  | | Mean | N | Std. Deviation | Std. Error Mean |
| Pair 1 | CavgPelvTilt | 10,4312 | 57 | 5,35323 | ,70905 |
|  | VavgPelvTilt | 9,5905 | 57 | 5,80151 | ,76843 |
| Pair 2 | CPeakHipExt | -9,6102 | 57 | 6,20520 | ,82190 |
|  | VPeakHipExt | -12,3905 | 57 | 7,09770 | ,94011 |
| Pair 3 | CPeakHipFLexTS | 38,8160 | 57 | 6,27495 | ,83114 |
|  | VPeakHipFLexTS | 33,3223 | 57 | 7,03721 | ,93210 |
| Pair 4 | CStancePhaseKneeFlexion | 26,1493 | 57 | 5,85970 | ,77614 |
|  | VStancePhasekneeflexion | 20,9474 | 57 | 6,87921 | ,91117 |
| Pair 5 | CkneeExtension_MS | 5,1540 | 57 | 4,16582 | ,55178 |
|  | VkneeExtension_MS | 4,3732 | 57 | 5,29252 | ,70101 |
| Pair 6 | CMaxKneeFlex_Swing | 69,8468 | 57 | 3,66237 | ,48509 |
|  | VMaxKneeFlexionSwing | 63,4228 | 57 | 5,87389 | ,77802 |
| Pair 7 | CPeakDorsalFlex | 10,7133 | 57 | 3,82686 | ,50688 |
|  | VPeakDorsalFlex | 14,4951 | 57 | 4,00519 | ,53050 |
| Pair 8 | CPeakPlantarFlex | -15,3474 | 57 | 5,60958 | ,74301 |
|  | VPeakPlantarFlex | -11,7658 | 57 | 5,17788 | ,68583 |

| **Paired Samples Correlations** | | | | |
| --- | --- | --- | --- | --- |
|  | | N | Correlation | Sig. |
| Pair 1 | CavgPelvTilt & VavgPelvTilt | 57 | ,773 | ,000 |
| Pair 2 | CPeakHipExt & VPeakHipExt | 57 | ,647 | ,000 |
| Pair 3 | CPeakHipFLexTS & VPeakHipFLexTS | 57 | ,558 | ,000 |
| Pair 4 | CStancePhaseKneeFlexion & VStancePhasekneeflexion | 57 | ,677 | ,000 |
| Pair 5 | CkneeExtension_MS & VkneeExtension_MS | 57 | ,484 | ,000 |
| Pair 6 | CMaxKneeFlex_Swing & VMaxKneeFlexionSwing | 57 | ,477 | ,000 |
| Pair 7 | CPeakDorsalFlex & VPeakDorsalFlex | 57 | ,499 | ,000 |
| Pair 8 | CPeakPlantarFlex & VPeakPlantarFlex | 57 | ,583 | ,000 |

| **Paired Samples Test** | | | | | | | | | |
| --- | --- | --- | --- | --- | --- | --- | --- | --- | --- |
|  | | Paired Differences | | | | | t | df | Sig. (2-tailed) |
|  |  | Mean | Std. Deviation | Std. Error Mean | 95% Confidence Interval of the Difference | |  |  |  |
|  |  |  |  |  | Lower | Upper |  |  |  |
| Pair 1 | CavgPelvTilt - VavgPelvTilt | ,84070 | 3,78134 | ,50085 | -,16262 | 1,84403 | 1,679 | 56 | ,099 |
| Pair 2 | CPeakHipExt - VPeakHipExt | 2,78035 | 5,64768 | ,74805 | 1,28182 | 4,27888 | 3,717 | 56 | ,000 |
| Pair 3 | CPeakHipFLexTS - VPeakHipFLexTS | 5,49368 | 6,29157 | ,83334 | 3,82431 | 7,16306 | 6,592 | 56 | ,000 |
| Pair 4 | CStancePhaseKneeFlexion - VStancePhasekneeflexion | 5,20193 | 5,20349 | ,68922 | 3,82126 | 6,58260 | 7,548 | 56 | ,000 |
| Pair 5 | CkneeExtension_MS - VkneeExtension_MS | ,78088 | 4,90242 | ,64934 | -,51991 | 2,08167 | 1,203 | 56 | ,234 |
| Pair 6 | CMaxKneeFlex_Swing - VMaxKneeFlexionSwing | 6,42404 | 5,23539 | ,69344 | 5,03490 | 7,81317 | 9,264 | 56 | ,000 |
| Pair 7 | CPeakDorsalFlex - VPeakDorsalFlex | -3,78175 | 3,92258 | ,51956 | -4,82256 | -2,74095 | -7,279 | 56 | ,000 |
| Pair 8 | CPeakPlantarFlex - VPeakPlantarFlex | -3,58158 | 4,94099 | ,65445 | -4,89260 | -2,27056 | -5,473 | 56 | ,000 |

- 1. **Peaks in GRFs**

| **Paired Samples Statistics** | | | | | |
| --- | --- | --- | --- | --- | --- |
|  | | Mean | N | Std. Deviation | Std. Error Mean |
| Pair 1 | ViconMaxPost | ,2041 | 56 | ,04594 | ,00614 |
|  | MaxPostCaren | ,1993 | 56 | ,04125 | ,00551 |
| Pair 2 | ViconMaxAnt | -,2150 | 56 | ,03974 | ,00531 |
|  | maxAntCaren | -,2089 | 56 | ,03060 | ,00409 |
| Pair 3 | Viconthough | ,7158 | 56 | ,08812 | ,01178 |
|  | thoughCaren | ,7199 | 56 | ,07562 | ,01010 |
| Pair 4 | ViconPeak2 | 1,1027 | 56 | ,08719 | ,01165 |
|  | Peak2Caren | 1,0785 | 56 | ,07734 | ,01034 |

| **Paired Samples Correlations** | | | | |
| --- | --- | --- | --- | --- |
|  | | N | Correlation | Sig. |
| Pair 1 | ViconMaxPost & MaxPostCaren | 56 | ,806 | ,000 |
| Pair 2 | ViconMaxAnt & maxAntCaren | 56 | ,755 | ,000 |
| Pair 3 | Viconthough & thoughCaren | 56 | ,795 | ,000 |
| Pair 4 | ViconPeak2 & Peak2Caren | 56 | ,801 | ,000 |

| **Paired Samples Test** | | | | | | | | | | |
| --- | --- | --- | --- | --- | --- | --- | --- | --- | --- | --- |
|  | | Paired Differences | | | | | t | df | Sig. (2-tailed) |  |
|  |  | Mean | Std. Deviation | Std. Error Mean | 95% Confidence Interval of the Difference | |  |  |  |  |
|  |  |  |  |  | Lower | Upper |  |  |  |  |
| Pair 1 | ViconMaxPost - MaxPostCaren | ,00487 | ,02755 | ,00368 | -,00251 | ,01224 | 1,322 | 55 | ,192 |  |
| Pair 2 | ViconMaxAnt - maxAntCaren | -,00603 | ,02607 | ,00348 | -,01301 | ,00095 | -1,731 | 55 | ,089 |  |
| Pair 3 | Viconthough - thoughCaren | -,00407 | ,05376 | ,00718 | -,01847 | ,01033 | -,566 | 55 | ,573 |  |
| Pair 4 | ViconPeak2 - Peak2Caren | ,02423 | ,05270 | ,00704 | ,01011 | ,03834 | 3,440 | 55 | ,001 |  |


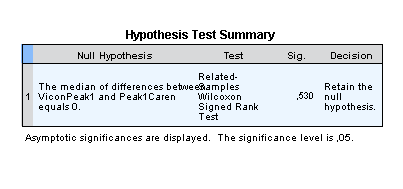


- 1. **Peaks in sagittal joint moments**

| **Paired Samples Statistics^a^** | | | | | |
| --- | --- | --- | --- | --- | --- |
|  | | Mean | N | Std. Deviation | Std. Error Mean |
| Pair 1 | PeakHipExtM1 | ,9705 | 56 | ,29065 | ,03884 |
|  | PeakHipExtM1Caren | ,6474 | 56 | ,17462 | ,02333 |
| Pair 2 | PeakHipFlexM | -,9453 | 56 | ,19370 | ,02588 |
|  | PeakHipFlexMCAREN | -,7357 | 56 | ,17771 | ,02375 |
| Pair 3 | PeakAnklePFMoment | 1,2690 | 56 | ,21206 | ,02834 |
|  | PeakAnklePFMCaren | 1,3094 | 56 | ,24780 | ,03311 |
| Pair 4 | PeakKneeExtMoment | ,5267 | 56 | ,35377 | ,04727 |
|  | PeakKneeExtCAREN | ,5102 | 56 | ,16743 | ,02237 |
|  | | | | | |

| **Paired Samples Correlations^a^** | | | | |
| --- | --- | --- | --- | --- |
|  | | N | Correlation | Sig. |
| Pair 1 | PeakHipExtM1 & PeakHipExtM1Caren | 56 | ,279 | ,037 |
| Pair 2 | PeakHipFlexM & PeakHipFlexMCAREN | 56 | ,611 | ,000 |
| Pair 3 | PeakAnklePFMoment & PeakAnklePFMCaren | 56 | ,870 | ,000 |
| Pair 4 | PeakKneeExtMoment & PeakKneeExtCAREN | 56 | ,185 | ,171 |
| \| **Paired Samples Test^a^** \| \| \| \| \| \| \| \| \| \| \| --- \| --- \| --- \| --- \| --- \| --- \| --- \| --- \| --- \| --- \| \|  \| \| Paired Differences \| \| \| \| \| t \| df \| Sig. (2-tailed) \| \| Mean \| Std. Deviation \| Std. Error Mean \| 95% Confidence Interval of the Difference \| \| \| Lower \| Upper \| \| Pair 1 \| PeakHipExtM1 - PeakHipExtM1Caren \| ,32309 \| ,29439 \| ,03934 \| ,24425 \| ,40193 \| 8,213 \| 55 \| ,000 \| \| Pair 2 \| PeakHipFlexM - PeakHipFlexMCAREN \| -,20954 \| ,16448 \| ,02198 \| -,25358 \| -,16549 \| -9,533 \| 55 \| ,000 \| \| Pair 3 \| PeakAnklePFMoment - PeakAnklePFMCaren \| -,04039 \| ,12227 \| ,01634 \| -,07314 \| -,00765 \| -2,472 \| 55 \| ,017 \| \| Pair 4 \| PeakKneeExtMoment - PeakKneeExtCAREN \| ,01650 \| ,36223 \| ,04841 \| -,08051 \| ,11351 \| ,341 \| 55 \| ,735 \| \|  \| \| \| \| \| \| \| \| \| \| | | | | |


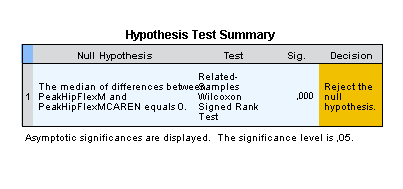


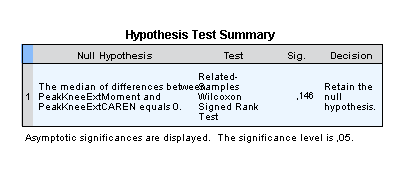


1. **Mean differences**
   1. **Mean difference in spatiotemporal parameters**

| **Descriptives** | | | | |
| --- | --- | --- | --- | --- |
|  | | | Statistic | Std. Error |
| MDspeed | Mean | | ,02354 | ,006611 |
|  | 95% Confidence Interval for Mean | Lower Bound | ,01030 |  |
|  |  | Upper Bound | ,03679 |  |
|  | 5% Trimmed Mean | | ,02184 |  |
|  | Median | | ,01352 |  |
|  | Variance | | ,002 |  |
|  | Std. Deviation | | ,049914 |  |
|  | Minimum | | -,070 |  |
|  | Maximum | | ,164 |  |
|  | Range | | ,234 |  |
|  | Interquartile Range | | ,049 |  |
|  | Skewness | | ,557 | ,316 |
|  | Kurtosis | | ,702 | ,623 |
| MDstridetime_R | Mean | | ,01877 | ,004636 |
|  | 95% Confidence Interval for Mean | Lower Bound | ,00948 |  |
|  |  | Upper Bound | ,02805 |  |
|  | 5% Trimmed Mean | | ,01870 |  |
|  | Median | | ,01840 |  |
|  | Variance | | ,001 |  |
|  | Std. Deviation | | ,035001 |  |
|  | Minimum | | -,062 |  |
|  | Maximum | | ,097 |  |
|  | Range | | ,159 |  |
|  | Interquartile Range | | ,048 |  |
|  | Skewness | | ,146 | ,316 |
|  | Kurtosis | | -,288 | ,623 |
| MDstance_R | Mean | | ,05147 | ,005998 |
|  | 95% Confidence Interval for Mean | Lower Bound | ,03945 |  |
|  |  | Upper Bound | ,06348 |  |
|  | 5% Trimmed Mean | | ,04666 |  |
|  | Median | | ,04613 |  |
|  | Variance | | ,002 |  |
|  | Std. Deviation | | ,045281 |  |
|  | Minimum | | -,024 |  |
|  | Maximum | | ,317 |  |
|  | Range | | ,341 |  |
|  | Interquartile Range | | ,031 |  |
|  | Skewness | | 3,934 | ,316 |
|  | Kurtosis | | 21,829 | ,623 |
| MDswing_R | Mean | | -,03235 | ,005101 |
|  | 95% Confidence Interval for Mean | Lower Bound | -,04257 |  |
|  |  | Upper Bound | -,02213 |  |
|  | 5% Trimmed Mean | | -,02807 |  |
|  | Median | | -,03000 |  |
|  | Variance | | ,001 |  |
|  | Std. Deviation | | ,038513 |  |
|  | Minimum | | -,241 |  |
|  | Maximum | | ,027 |  |
|  | Range | | ,269 |  |
|  | Interquartile Range | | ,031 |  |
|  | Skewness | | -3,253 | ,316 |
|  | Kurtosis | | 15,916 | ,623 |
| MDsteplength_R | Mean | | ,02210 | ,003727 |
|  | 95% Confidence Interval for Mean | Lower Bound | ,01463 |  |
|  |  | Upper Bound | ,02957 |  |
|  | 5% Trimmed Mean | | ,02153 |  |
|  | Median | | ,02164 |  |
|  | Variance | | ,001 |  |
|  | Std. Deviation | | ,028140 |  |
|  | Minimum | | -,034 |  |
|  | Maximum | | ,099 |  |
|  | Range | | ,133 |  |
|  | Interquartile Range | | ,035 |  |
|  | Skewness | | ,302 | ,316 |
|  | Kurtosis | | ,372 | ,623 |
| MDstepwidth_R | Mean | | -,03292 | ,004706 |
|  | 95% Confidence Interval for Mean | Lower Bound | -,04234 |  |
|  |  | Upper Bound | -,02349 |  |
|  | 5% Trimmed Mean | | -,03251 |  |
|  | Median | | -,02743 |  |
|  | Variance | | ,001 |  |
|  | Std. Deviation | | ,035527 |  |
|  | Minimum | | -,125 |  |
|  | Maximum | | ,068 |  |
|  | Range | | ,193 |  |
|  | Interquartile Range | | ,038 |  |
|  | Skewness | | -,201 | ,316 |
|  | Kurtosis | | ,850 | ,623 |

- 1. **Mean difference in sagittal joint angles**

| **Descriptives** | | | | |
| --- | --- | --- | --- | --- |
|  | | | Statistic | Std. Error |
| MD_PelvicTitlt | Mean | | -,8407 | ,50085 |
|  | 95% Confidence Interval for Mean | Lower Bound | -1,8440 |  |
|  |  | Upper Bound | ,1626 |  |
|  | 5% Trimmed Mean | | -,9513 |  |
|  | Median | | -,8500 |  |
|  | Variance | | 14,299 |  |
|  | Std. Deviation | | 3,78134 |  |
|  | Minimum | | -11,62 |  |
|  | Maximum | | 14,70 |  |
|  | Range | | 26,32 |  |
|  | Interquartile Range | | 3,73 |  |
|  | Skewness | | ,878 | ,316 |
|  | Kurtosis | | 4,752 | ,623 |
| MD_HipExtTS | Mean | | -2,7804 | ,74805 |
|  | 95% Confidence Interval for Mean | Lower Bound | -4,2789 |  |
|  |  | Upper Bound | -1,2818 |  |
|  | 5% Trimmed Mean | | -2,3777 |  |
|  | Median | | -1,5800 |  |
|  | Variance | | 31,896 |  |
|  | Std. Deviation | | 5,64768 |  |
|  | Minimum | | -23,38 |  |
|  | Maximum | | 8,20 |  |
|  | Range | | 31,58 |  |
|  | Interquartile Range | | 5,64 |  |
|  | Skewness | | -1,442 | ,316 |
|  | Kurtosis | | 3,551 | ,623 |
| MD_HipFlexTS | Mean | | -5,4937 | ,83334 |
|  | 95% Confidence Interval for Mean | Lower Bound | -7,1631 |  |
|  |  | Upper Bound | -3,8243 |  |
|  | 5% Trimmed Mean | | -5,0249 |  |
|  | Median | | -4,4200 |  |
|  | Variance | | 39,584 |  |
|  | Std. Deviation | | 6,29157 |  |
|  | Minimum | | -27,42 |  |
|  | Maximum | | 4,89 |  |
|  | Range | | 32,31 |  |
|  | Interquartile Range | | 6,42 |  |
|  | Skewness | | -1,464 | ,316 |
|  | Kurtosis | | 3,457 | ,623 |
| MD_SPKF | Mean | | -5,2019 | ,68922 |
|  | 95% Confidence Interval for Mean | Lower Bound | -6,5826 |  |
|  |  | Upper Bound | -3,8213 |  |
|  | 5% Trimmed Mean | | -4,9915 |  |
|  | Median | | -4,3900 |  |
|  | Variance | | 27,076 |  |
|  | Std. Deviation | | 5,20349 |  |
|  | Minimum | | -26,43 |  |
|  | Maximum | | 6,11 |  |
|  | Range | | 32,54 |  |
|  | Interquartile Range | | 6,38 |  |
|  | Skewness | | -1,076 | ,316 |
|  | Kurtosis | | 3,965 | ,623 |
| MD_KneeExtMS | Mean | | -,7809 | ,64934 |
|  | 95% Confidence Interval for Mean | Lower Bound | -2,0817 |  |
|  |  | Upper Bound | ,5199 |  |
|  | 5% Trimmed Mean | | -,5850 |  |
|  | Median | | -,5400 |  |
|  | Variance | | 24,034 |  |
|  | Std. Deviation | | 4,90242 |  |
|  | Minimum | | -17,82 |  |
|  | Maximum | | 13,31 |  |
|  | Range | | 31,13 |  |
|  | Interquartile Range | | 4,56 |  |
|  | Skewness | | -,669 | ,316 |
|  | Kurtosis | | 3,417 | ,623 |
| MD_maxKneeFlex | Mean | | -6,4240 | ,69344 |
|  | 95% Confidence Interval for Mean | Lower Bound | -7,8132 |  |
|  |  | Upper Bound | -5,0349 |  |
|  | 5% Trimmed Mean | | -5,9660 |  |
|  | Median | | -6,2600 |  |
|  | Variance | | 27,409 |  |
|  | Std. Deviation | | 5,23539 |  |
|  | Minimum | | -30,03 |  |
|  | Maximum | | 2,80 |  |
|  | Range | | 32,83 |  |
|  | Interquartile Range | | 5,08 |  |
|  | Skewness | | -1,966 | ,316 |
|  | Kurtosis | | 7,338 | ,623 |
| MD_peakDF | Mean | | 3,7818 | ,51956 |
|  | 95% Confidence Interval for Mean | Lower Bound | 2,7410 |  |
|  |  | Upper Bound | 4,8226 |  |
|  | 5% Trimmed Mean | | 4,0595 |  |
|  | Median | | 4,2700 |  |
|  | Variance | | 15,387 |  |
|  | Std. Deviation | | 3,92258 |  |
|  | Minimum | | -14,92 |  |
|  | Maximum | | 10,13 |  |
|  | Range | | 25,05 |  |
|  | Interquartile Range | | 4,11 |  |
|  | Skewness | | -1,962 | ,316 |
|  | Kurtosis | | 8,101 | ,623 |
| MD_peakPF | Mean | | 3,5816 | ,65445 |
|  | 95% Confidence Interval for Mean | Lower Bound | 2,2706 |  |
|  |  | Upper Bound | 4,8926 |  |
|  | 5% Trimmed Mean | | 3,7427 |  |
|  | Median | | 4,1500 |  |
|  | Variance | | 24,413 |  |
|  | Std. Deviation | | 4,94099 |  |
|  | Minimum | | -14,30 |  |
|  | Maximum | | 15,44 |  |
|  | Range | | 29,74 |  |
|  | Interquartile Range | | 7,02 |  |
|  | Skewness | | -,757 | ,316 |
|  | Kurtosis | | 2,072 | ,623 |

- 1. **Mean difference in GRFs**

| **Descriptives^a^** | | | | |
| --- | --- | --- | --- | --- |
|  | | | Statistic | Std. Error |
| MD_maxpost | Mean | | ,0073 | ,00277 |
|  | 95% Confidence Interval for Mean | Lower Bound | ,0017 |  |
|  |  | Upper Bound | ,0128 |  |
|  | 5% Trimmed Mean | | ,0072 |  |
|  | Median | | ,0061 |  |
|  | Variance | | ,000 |  |
|  | Std. Deviation | | ,02094 |  |
|  | Minimum | | -,05 |  |
|  | Maximum | | ,06 |  |
|  | Range | | ,10 |  |
|  | Interquartile Range | | ,03 |  |
|  | Skewness | | ,185 | ,316 |
|  | Kurtosis | | ,188 | ,623 |
| MD_ant | Mean | | -,0044 | ,00316 |
|  | 95% Confidence Interval for Mean | Lower Bound | -,0107 |  |
|  |  | Upper Bound | ,0020 |  |
|  | 5% Trimmed Mean | | -,0038 |  |
|  | Median | | -,0018 |  |
|  | Variance | | ,001 |  |
|  | Std. Deviation | | ,02388 |  |
|  | Minimum | | -,06 |  |
|  | Maximum | | ,04 |  |
|  | Range | | ,11 |  |
|  | Interquartile Range | | ,03 |  |
|  | Skewness | | -,360 | ,316 |
|  | Kurtosis | | -,158 | ,623 |
| MD_peak1 | Mean | | -,0093 | ,00873 |
|  | 95% Confidence Interval for Mean | Lower Bound | -,0268 |  |
|  |  | Upper Bound | ,0082 |  |
|  | 5% Trimmed Mean | | -,0111 |  |
|  | Median | | -,0029 |  |
|  | Variance | | ,004 |  |
|  | Std. Deviation | | ,06592 |  |
|  | Minimum | | -,18 |  |
|  | Maximum | | ,21 |  |
|  | Range | | ,39 |  |
|  | Interquartile Range | | ,07 |  |
|  | Skewness | | ,402 | ,316 |
|  | Kurtosis | | 2,649 | ,623 |
| MD_though | Mean | | -,0019 | ,00557 |
|  | 95% Confidence Interval for Mean | Lower Bound | -,0131 |  |
|  |  | Upper Bound | ,0092 |  |
|  | 5% Trimmed Mean | | -,0018 |  |
|  | Median | | ,0000 |  |
|  | Variance | | ,002 |  |
|  | Std. Deviation | | ,04205 |  |
|  | Minimum | | -,09 |  |
|  | Maximum | | ,08 |  |
|  | Range | | ,17 |  |
|  | Interquartile Range | | ,05 |  |
|  | Skewness | | -,056 | ,316 |
|  | Kurtosis | | -,481 | ,623 |
| MD_peak2 | Mean | | ,0260 | ,00732 |
|  | 95% Confidence Interval for Mean | Lower Bound | ,0114 |  |
|  |  | Upper Bound | ,0407 |  |
|  | 5% Trimmed Mean | | ,0243 |  |
|  | Median | | ,0137 |  |
|  | Variance | | ,003 |  |
|  | Std. Deviation | | ,05525 |  |
|  | Minimum | | -,12 |  |
|  | Maximum | | ,20 |  |
|  | Range | | ,32 |  |
|  | Interquartile Range | | ,08 |  |
|  | Skewness | | ,555 | ,316 |
|  | Kurtosis | | 1,157 | ,623 |
|  | | | | |

- 1. **Mean difference in sagittal joint moments**

| **Descriptives^a^** | | | | |
| --- | --- | --- | --- | --- |
|  | | | Statistic | Std. Error |
| MD_HipExt | Mean | | ,3231 | ,03934 |
|  | 95% Confidence Interval for Mean | Lower Bound | ,2443 |  |
|  |  | Upper Bound | ,4019 |  |
|  | 5% Trimmed Mean | | ,3213 |  |
|  | Median | | ,3425 |  |
|  | Variance | | ,087 |  |
|  | Std. Deviation | | ,29439 |  |
|  | Minimum | | -,41 |  |
|  | Maximum | | 1,17 |  |
|  | Range | | 1,58 |  |
|  | Interquartile Range | | ,35 |  |
|  | Skewness | | ,076 | ,319 |
|  | Kurtosis | | 1,093 | ,628 |
| MD_HipFlex | Mean | | -,2095 | ,02198 |
|  | 95% Confidence Interval for Mean | Lower Bound | -,2536 |  |
|  |  | Upper Bound | -,1655 |  |
|  | 5% Trimmed Mean | | -,2108 |  |
|  | Median | | -,2070 |  |
|  | Variance | | ,027 |  |
|  | Std. Deviation | | ,16448 |  |
|  | Minimum | | -,56 |  |
|  | Maximum | | ,18 |  |
|  | Range | | ,74 |  |
|  | Interquartile Range | | ,20 |  |
|  | Skewness | | -,009 | ,319 |
|  | Kurtosis | | ,132 | ,628 |
| MD_KneeExtMoment | Mean | | ,0165 | ,04841 |
|  | 95% Confidence Interval for Mean | Lower Bound | -,0805 |  |
|  |  | Upper Bound | ,1135 |  |
|  | 5% Trimmed Mean | | -,0246 |  |
|  | Median | | -,0217 |  |
|  | Variance | | ,131 |  |
|  | Std. Deviation | | ,36223 |  |
|  | Minimum | | -,56 |  |
|  | Maximum | | 1,75 |  |
|  | Range | | 2,32 |  |
|  | Interquartile Range | | ,27 |  |
|  | Skewness | | 2,929 | ,319 |
|  | Kurtosis | | 12,158 | ,628 |
| MD_PFmoment | Mean | | -,0404 | ,01634 |
|  | 95% Confidence Interval for Mean | Lower Bound | -,0731 |  |
|  |  | Upper Bound | -,0076 |  |
|  | 5% Trimmed Mean | | -,0413 |  |
|  | Median | | -,0415 |  |
|  | Variance | | ,015 |  |
|  | Std. Deviation | | ,12227 |  |
|  | Minimum | | -,28 |  |
|  | Maximum | | ,22 |  |
|  | Range | | ,50 |  |
|  | Interquartile Range | | ,19 |  |
|  | Skewness | | ,228 | ,319 |
|  | Kurtosis | | -,767 | ,628 |
|  | | | | |

1. **Statistical parametric mapping, using a two-tailed paired t-test, was performed to compare waveforms of sagittal angles, moments and GRFs between the two approaches.**

SPM analyses were implemented using the open-source spm1d code (v.M0.1, [www.spm1d.org](http://www.spm1d.org)) in MATLAB (Mathworks, Natick, MA, USA).

The results are plotted in Figure 1, 2 and 3, presented in the manuscript.

1. **Root mean square differences were calculated.** See additional excel file.

| **Descriptives** | | | | |
| --- | --- | --- | --- | --- |
|  | | | Statistic | Std. Error |
| RMSE_sagHip | Mean | | 7,9139 | ,57710 |
|  | 95% Confidence Interval for Mean | Lower Bound | 6,7578 |  |
|  |  | Upper Bound | 9,0700 |  |
|  | 5% Trimmed Mean | | 7,3291 |  |
|  | Median | | 6,4051 |  |
|  | Variance | | 18,984 |  |
|  | Std. Deviation | | 4,35703 |  |
|  | Minimum | | 3,14 |  |
|  | Maximum | | 25,38 |  |
|  | Range | | 22,23 |  |
|  | Interquartile Range | | 3,43 |  |
|  | Skewness | | 2,427 | ,316 |
|  | Kurtosis | | 6,471 | ,623 |
| RMSE_sagknee | Mean | | 9,0352 | ,45554 |
|  | 95% Confidence Interval for Mean | Lower Bound | 8,1227 |  |
|  |  | Upper Bound | 9,9478 |  |
|  | 5% Trimmed Mean | | 8,6735 |  |
|  | Median | | 8,1946 |  |
|  | Variance | | 11,829 |  |
|  | Std. Deviation | | 3,43928 |  |
|  | Minimum | | 3,37 |  |
|  | Maximum | | 27,21 |  |
|  | Range | | 23,84 |  |
|  | Interquartile Range | | 3,21 |  |
|  | Skewness | | 2,982 | ,316 |
|  | Kurtosis | | 13,947 | ,623 |
| RMSE_sagANkle | Mean | | 5,4874 | ,28082 |
|  | 95% Confidence Interval for Mean | Lower Bound | 4,9248 |  |
|  |  | Upper Bound | 6,0499 |  |
|  | 5% Trimmed Mean | | 5,3115 |  |
|  | Median | | 5,0300 |  |
|  | Variance | | 4,495 |  |
|  | Std. Deviation | | 2,12016 |  |
|  | Minimum | | 2,04 |  |
|  | Maximum | | 14,27 |  |
|  | Range | | 12,23 |  |
|  | Interquartile Range | | 2,28 |  |
|  | Skewness | | 1,694 | ,316 |
|  | Kurtosis | | 4,503 | ,623 |
| RMSE_pelvTilt | Mean | | 2,9824 | ,33919 |
|  | 95% Confidence Interval for Mean | Lower Bound | 2,3029 |  |
|  |  | Upper Bound | 3,6619 |  |
|  | 5% Trimmed Mean | | 2,6452 |  |
|  | Median | | 2,3470 |  |
|  | Variance | | 6,558 |  |
|  | Std. Deviation | | 2,56086 |  |
|  | Minimum | | ,58 |  |
|  | Maximum | | 14,72 |  |
|  | Range | | 14,13 |  |
|  | Interquartile Range | | 2,66 |  |
|  | Skewness | | 2,545 | ,316 |
|  | Kurtosis | | 8,636 | ,623 |
| OCRMSD_hip_X | Mean | | 2,6814 | ,18919 |
|  | 95% Confidence Interval for Mean | Lower Bound | 2,3024 |  |
|  |  | Upper Bound | 3,0604 |  |
|  | 5% Trimmed Mean | | 2,6366 |  |
|  | Median | | 2,2922 |  |
|  | Variance | | 2,040 |  |
|  | Std. Deviation | | 1,42833 |  |
|  | Minimum | | ,15 |  |
|  | Maximum | | 6,03 |  |
|  | Range | | 5,89 |  |
|  | Interquartile Range | | 2,10 |  |
|  | Skewness | | ,519 | ,316 |
|  | Kurtosis | | -,560 | ,623 |
| OCRMSD_knee_X | Mean | | 4,3939 | ,25119 |
|  | 95% Confidence Interval for Mean | Lower Bound | 3,8907 |  |
|  |  | Upper Bound | 4,8971 |  |
|  | 5% Trimmed Mean | | 4,3394 |  |
|  | Median | | 4,0958 |  |
|  | Variance | | 3,597 |  |
|  | Std. Deviation | | 1,89648 |  |
|  | Minimum | | ,92 |  |
|  | Maximum | | 8,66 |  |
|  | Range | | 7,74 |  |
|  | Interquartile Range | | 3,10 |  |
|  | Skewness | | ,466 | ,316 |
|  | Kurtosis | | -,772 | ,623 |
| OCRMSD_ankle_X | Mean | | 2,0026 | ,13068 |
|  | 95% Confidence Interval for Mean | Lower Bound | 1,7409 |  |
|  |  | Upper Bound | 2,2644 |  |
|  | 5% Trimmed Mean | | 1,9385 |  |
|  | Median | | 1,9500 |  |
|  | Variance | | ,973 |  |
|  | Std. Deviation | | ,98660 |  |
|  | Minimum | | ,44 |  |
|  | Maximum | | 5,38 |  |
|  | Range | | 4,94 |  |
|  | Interquartile Range | | 1,28 |  |
|  | Skewness | | 1,017 | ,316 |
|  | Kurtosis | | 1,794 | ,623 |
| OCRMSD_pelvtilt_X | Mean | | ,1692 | ,02136 |
|  | 95% Confidence Interval for Mean | Lower Bound | ,1264 |  |
|  |  | Upper Bound | ,2119 |  |
|  | 5% Trimmed Mean | | ,1501 |  |
|  | Median | | ,1326 |  |
|  | Variance | | ,026 |  |
|  | Std. Deviation | | ,16124 |  |
|  | Minimum | | ,01 |  |
|  | Maximum | | ,73 |  |
|  | Range | | ,72 |  |
|  | Interquartile Range | | ,13 |  |
|  | Skewness | | 2,005 | ,316 |
|  | Kurtosis | | 3,842 | ,623 |

| **Descriptives** | | | | |
| --- | --- | --- | --- | --- |
|  | | | Statistic | Std. Error |
| RMSE_AnkleMoment | Mean | | ,1175 | ,00520 |
|  | 95% Confidence Interval for Mean | Lower Bound | ,1071 |  |
|  |  | Upper Bound | ,1279 |  |
|  | 5% Trimmed Mean | | ,1153 |  |
|  | Median | | ,1164 |  |
|  | Variance | | ,002 |  |
|  | Std. Deviation | | ,03893 |  |
|  | Minimum | | ,05 |  |
|  | Maximum | | ,25 |  |
|  | Range | | ,20 |  |
|  | Interquartile Range | | ,05 |  |
|  | Skewness | | ,942 | ,319 |
|  | Kurtosis | | 1,852 | ,628 |
| RMSE_KneeMoment | Mean | | ,1815 | ,01398 |
|  | 95% Confidence Interval for Mean | Lower Bound | ,1535 |  |
|  |  | Upper Bound | ,2095 |  |
|  | 5% Trimmed Mean | | ,1659 |  |
|  | Median | | ,1633 |  |
|  | Variance | | ,011 |  |
|  | Std. Deviation | | ,10463 |  |
|  | Minimum | | ,07 |  |
|  | Maximum | | ,68 |  |
|  | Range | | ,61 |  |
|  | Interquartile Range | | ,06 |  |
|  | Skewness | | 3,750 | ,319 |
|  | Kurtosis | | 16,125 | ,628 |
| RMSE_HipMoment | Mean | | ,2465 | ,01092 |
|  | 95% Confidence Interval for Mean | Lower Bound | ,2246 |  |
|  |  | Upper Bound | ,2683 |  |
|  | 5% Trimmed Mean | | ,2403 |  |
|  | Median | | ,2305 |  |
|  | Variance | | ,007 |  |
|  | Std. Deviation | | ,08172 |  |
|  | Minimum | | ,12 |  |
|  | Maximum | | ,61 |  |
|  | Range | | ,49 |  |
|  | Interquartile Range | | ,07 |  |
|  | Skewness | | 1,848 | ,319 |
|  | Kurtosis | | 5,830 | ,628 |
| RMSE_VertGRF | Mean | | ,0759 | ,00262 |
|  | 95% Confidence Interval for Mean | Lower Bound | ,0707 |  |
|  |  | Upper Bound | ,0812 |  |
|  | 5% Trimmed Mean | | ,0755 |  |
|  | Median | | ,0750 |  |
|  | Variance | | ,000 |  |
|  | Std. Deviation | | ,01963 |  |
|  | Minimum | | ,04 |  |
|  | Maximum | | ,12 |  |
|  | Range | | ,08 |  |
|  | Interquartile Range | | ,03 |  |
|  | Skewness | | ,231 | ,319 |
|  | Kurtosis | | -,633 | ,628 |
| RMSE_APGRF | Mean | | ,0192 | ,00088 |
|  | 95% Confidence Interval for Mean | Lower Bound | ,0174 |  |
|  |  | Upper Bound | ,0209 |  |
|  | 5% Trimmed Mean | | ,0186 |  |
|  | Median | | ,0170 |  |
|  | Variance | | ,000 |  |
|  | Std. Deviation | | ,00656 |  |
|  | Minimum | | ,01 |  |
|  | Maximum | | ,05 |  |
|  | Range | | ,04 |  |
|  | Interquartile Range | | ,01 |  |
|  | Skewness | | 1,900 | ,319 |
|  | Kurtosis | | 5,519 | ,628 |
